# Neutrophil-associated extracellular DNA pathways in vitiligo: compartment-specific changes following combined fractional CO_2_ laser and NB-UVB therapy

**DOI:** 10.3389/fimmu.2026.1897885

**Published:** 2026-09-01

**Authors:** Joanna Czerwińska, Alicja Frączek, Agnieszka Owczarczyk-Saczonek

**Affiliations:** Department of Dermatology, Sexually Transmitted Diseases and Clinical Immunology, School of Medicine, Collegium Medicum, University of Warmia and Mazury in Olsztyn, Olsztyn, Poland

**Keywords:** DNase I, laser, MPO-DNA complex, NETs, neutrophils, PAD-4, vitiligo

## Abstract

**Background:**

Vitiligo is a chronic inflammatory depigmenting skin disorder characterized by progressive loss of functional melanocytes, in which adaptive immune mechanisms, particularly T-cell-mediated responses, play a well-established role. Emerging evidence suggests that innate immune pathways, including neutrophil activation, oxidative stress, and dysregulation of neutrophil extracellular trap (NET)-associated processes, may also contribute to disease-associated inflammation.

**Methods:**

This study investigated NET-associated markers (MPO–DNA complexes, PAD-4, and DNase I) in patients with vitiligo before and after six months of combined fractional CO_2_ laser and narrowband UVB therapy.

**Results:**

At baseline, vitiligo patients exhibited elevated circulating MPO–DNA complexes and DNase I levels compared with healthy controls, whereas systemic PAD-4 levels were decreased. Lesional skin demonstrated increased expression of MPO, PAD-4, and DNase I compared with healthy skin. After six months of combined therapy, serum DNase I levels increased further, whereas lesional skin showed reduced MPO and PAD-4 expression together with a further increase in DNase I expression.

**Conclusions:**

These findings may indicate altered extracellular DNA handling and compartment-specific modulation of NET-associated pathways in vitiligo, although the functional significance of these changes remains unclear. Overall, the results suggest that NET-associated parameters may reflect local and systemic inflammatory changes associated with vitiligo and with treatment-related changes. Further studies are warranted to clarify the underlying mechanisms, validate these observations in larger longitudinal cohorts, and determine their potential relevance as indicators of disease-associated inflammatory activity.

## 1 Introduction

Vitiligo is a chronic inflammatory disorder characterized by progressive loss of functional epidermal melanocytes, resulting in characteristic non-scaling depigmented macules. The disease has a global prevalence of up to 2% and affects approximately 0.4% of European Caucasian populations (1). An international consensus established segmental vitiligo as a distinct clinical entity, whereas non-segmental vitiligo encompasses the remaining disease forms (2). Although genetic susceptibility contributes significantly to disease risk, vitiligo is currently recognized as a complex inflammatory and autoimmune disorder involving dysregulation of both adaptive and innate immune responses. In particular, interferon-gamma (IFN-γ)-producing CD8+ T cells and downstream IFN-γ-dependent chemokine pathways play a central role in melanocyte loss and disease progression (3, 4).

Beyond adaptive immunity, increasing evidence indicates that innate immune mechanisms may also contribute to the inflammatory environment observed in vitiligo. Neutrophils may participate in inflammatory amplification, as suggested by alterations in systemic inflammatory parameters, including the neutrophil-to-lymphocyte ratio (NLR) and C-reactive protein (CRP) levels (5, 6). Peripheral blood studies have demonstrated altered activation profiles of neutrophils, B cells, and monocytes in patients with vitiligo, reflecting complex interactions between immune, cytokine, and neuroendocrine pathways (7, 8). Furthermore, calprotectin, a neutrophil-derived inflammatory mediator, has been reported to be elevated in vitiligo and proposed as a potential indicator of inflammatory activity (9, 10). These findings suggest that neutrophil-associated pathways may contribute to shaping the inflammatory microenvironment accompanying vitiligo, although their functional role in disease development remains incompletely understood.

Neutrophil extracellular traps (NETs) represent an important mechanism by which neutrophils regulate inflammation through the release of extracellular DNA structures containing antimicrobial and inflammatory proteins (11). Under physiological conditions, NET formation contributes to host defense and resolution of inflammation; however, excessive NET formation or impaired regulation of NET-associated processes has been implicated in several autoimmune and inflammatory diseases. In vitiligo, where oxidative stress and innate immune activation contribute to melanocyte dysfunction and loss, investigation of NET-associated pathways may provide additional insight into inflammatory mechanisms involved in disease-associated tissue alterations (12).

Narrowband UV-B (NB-UVB) remains the standard phototherapeutic approach for generalized vitiligo due to its immunomodulatory effects, including regulation of inflammatory responses and promotion of melanocyte regeneration and migration (13). Fractional CO_2_ laser therapy has emerged as a potential adjunctive treatment, particularly when combined with NB-UVB, as controlled microthermal injury may promote epidermal remodeling, tissue regeneration, and modulation of the local inflammatory microenvironment. However, fractional CO_2_ laser is not considered a first-line treatment for vitiligo, and the immunological mechanisms underlying its potential therapeutic effects remain incompletely characterized. Clinical studies have suggested improved repigmentation outcomes following combined fractional CO_2_ laser and NB-UVB therapy; however, the biological processes responsible for these effects remain insufficiently understood (14, 15). Therefore, evaluation of NET-associated changes following this therapeutic approach may provide additional insight into the interaction between tissue remodeling and inflammatory regulation in vitiligo.

The present study aimed to investigate NET-associated processes in vitiligo, with particular emphasis on differences between systemic and cutaneous compartments. We assessed serum levels of MPO–DNA complexes, PAD-4, and DNase I together with cutaneous expression of MPO, PAD-4, and DNase I to determine whether vitiligo is associated with alterations in NET-associated pathways. Furthermore, we evaluated changes in these parameters after six months of combined fractional CO_2_ laser and narrowband UV-B therapy.

We hypothesized that vitiligo may be associated with compartment-specific alterations in NET-associated pathways, reflecting differences between systemic circulation and the local skin microenvironment. Additionally, we explored whether therapeutic intervention is associated with modulation of neutrophil-associated inflammatory processes and extracellular DNA metabolism.

## 2 Results

### 2.1 Disease activity assessment - vitiligo area scoring index and body surface area

Assessment of disease activity using the VASI and BSA clinical scales demonstrated a significant reduction in both VASI (p = 0.001) and BSA (p = 0.003) scores (**Table 1**). No significant correlations were found between the clinical scores and the analyzed serum or cutaneous parameters. Individual patient data are provided as dot plots in **Supplementary Figure 1**.

**Table 1:** Vitiligo area scoring index (VASI) and body surface area (BSA) values at baseline (T1) and after treatment (T2).

Vitiligo n=17	T1 – week 0		T2 – after 6 months	
	VASI	BSA (%)	VASI	BSA (%)
mean ± SEM	3.03 ± 0.64	3.53 ± 0.71	2.35 ± 0.54	2.77 ± 0.59
median	3	3.5	1.76	2.49
min/max	0.46/10.35	0.5/11.5	0.25/8.75	0.25/9.5

### 2.2 Blood assessment

First, peripheral blood samples obtained from all study participants were analyzed to determine neutrophil counts and C-reactive protein (CRP) levels as markers of systemic inflammation. In the vitiligo group, mean neutrophil count and CRP level were 4.33 ± 0.39 × 10³/µL and 1.95 ± 0.25 mg/L, respectively, compared with 3.94 ± 0.22 × 10³/µL and 0.79 ± 0.05 mg/L in healthy controls. Statistical comparison between the groups revealed no significant difference in neutrophil counts (p = 0.356) and a significant difference in CRP levels (p = 0.003). Despite these differences, both parameters remained within the reference ranges (1.8–7.7 × 10³/µL and <5.0 mg/L, respectively). Individual data are presented in **Supplementary Figure 2**.

### 2.3 Serum NET-associated markers before (T1) and after therapy (T2)

Next, serum concentrations of key NETosis-related markers were analyzed, including MPO–DNA complexes as indicators of NET release, PAD-4 as an enzyme essential for NET formation, and DNase I as a mediator of NET degradation. Additionally, the correlations between these parameters and clinical or inflammatory indices were evaluated.

**Figure 1:**
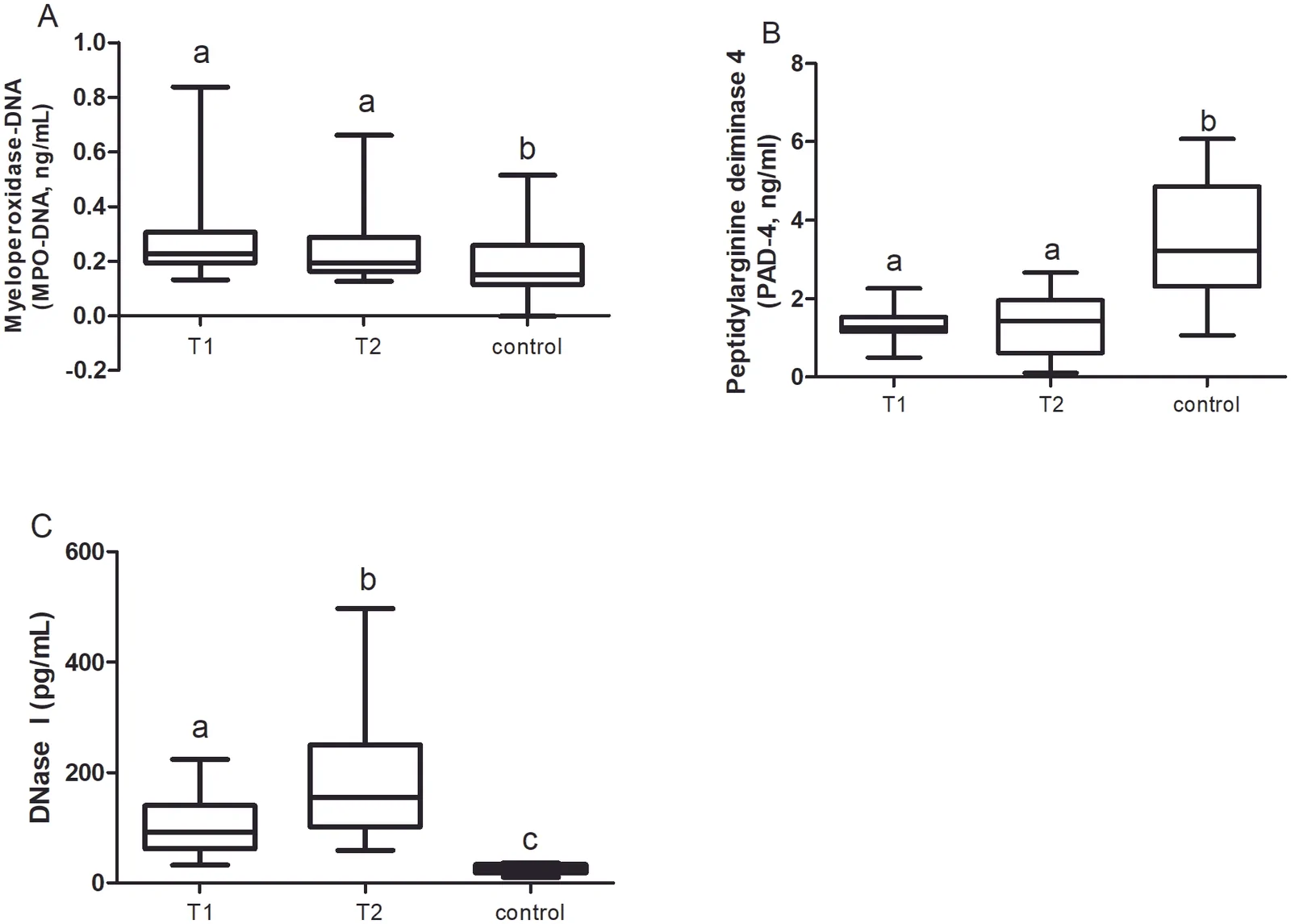
The mean (± SEM) level of **(A)** myeloperoxidase-DNA complexes (MPO, ng/mL), **(B)** peptidylarginine deiminase 4 (PAD-4, ng/mL) and **(C)** DNase I (pg/mL) in the blood serum of the patients with vitiligo (n=17) before (T1) and after 6 months of laser therapy (T2) in comparison to the control group (n = 31). The Wilcoxon signed-rank test was used to compare two time points, and the Kruskal-Wallis test was used to compare each of the studied groups with the control group. Whiskers indicate the range (min to max). Different letters indicate statistically significant differences between groups (p < 0.05), whereas groups sharing the same letter are not significantly different.

Elevated concentrations (p<0.05) of MPO–DNA and DNase I were observed at baseline (T1) compared with healthy controls (0.29 ± 0.05 ng/mL vs. 0.19 ± 0.02 and 105,18 ± 13.4 pg/mL vs. 25.96 ± 1.33 pg/mL, respectively; **Figures 1A, C**). In contrast, PAD-4 levels were significantly lower in vitiligo patients before therapy than in healthy controls (1.34 ± 0.09 ng/mL vs. 3.37 ± 0.26 ng/mL; p<0.05; **Figure 1B**). At the second study time point (T2), significant changes were observed exclusively for DNase I, which showed a further increase (T1 vs T2, 105,18 ± 13.4 pg/mL vs. 180.96 ± 28.09 pg/mL, p<0.05; **Figure 1C**). No significant correlations were found between these markers and disease activity (p>0.05).

### 2.4 Expression of MPO, PAD4, and DNase I in lesional and perilesional skin before (T1) and after therapy (T2)

**Figure 2:**
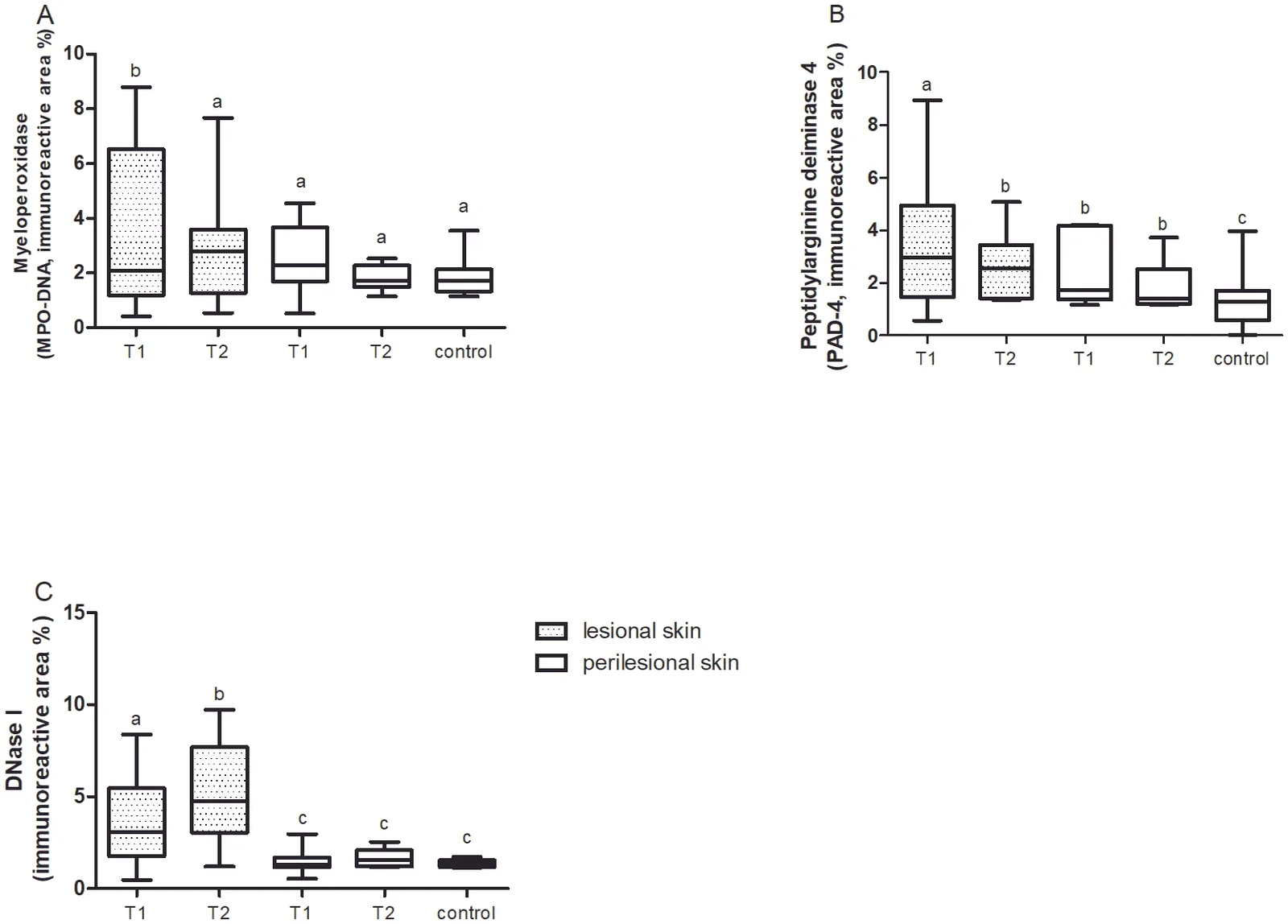
Immunoreactive area (means ± SEM) of **(A)** myeloperoxidase (MPO), **(B)** peptidylarginine deiminase 4 (PAD-4) and **(C)** DNase I in lesional (n=17) and non-lesional (n=7) skin of the patients with vitiligo before (T1) and after 6 months of laser therapy (T2) in comparison to the control group (n = 12). The Wilcoxon signed-rank test was used to compare two time points, and the Kruskal-Wallis test was used to compare each of the studied groups with the control group. Whiskers indicate the range (min to max). Different letters indicate statistically significant differences between groups (p < 0.05), whereas groups sharing the same letter are not significantly different.

**Figure 3:**
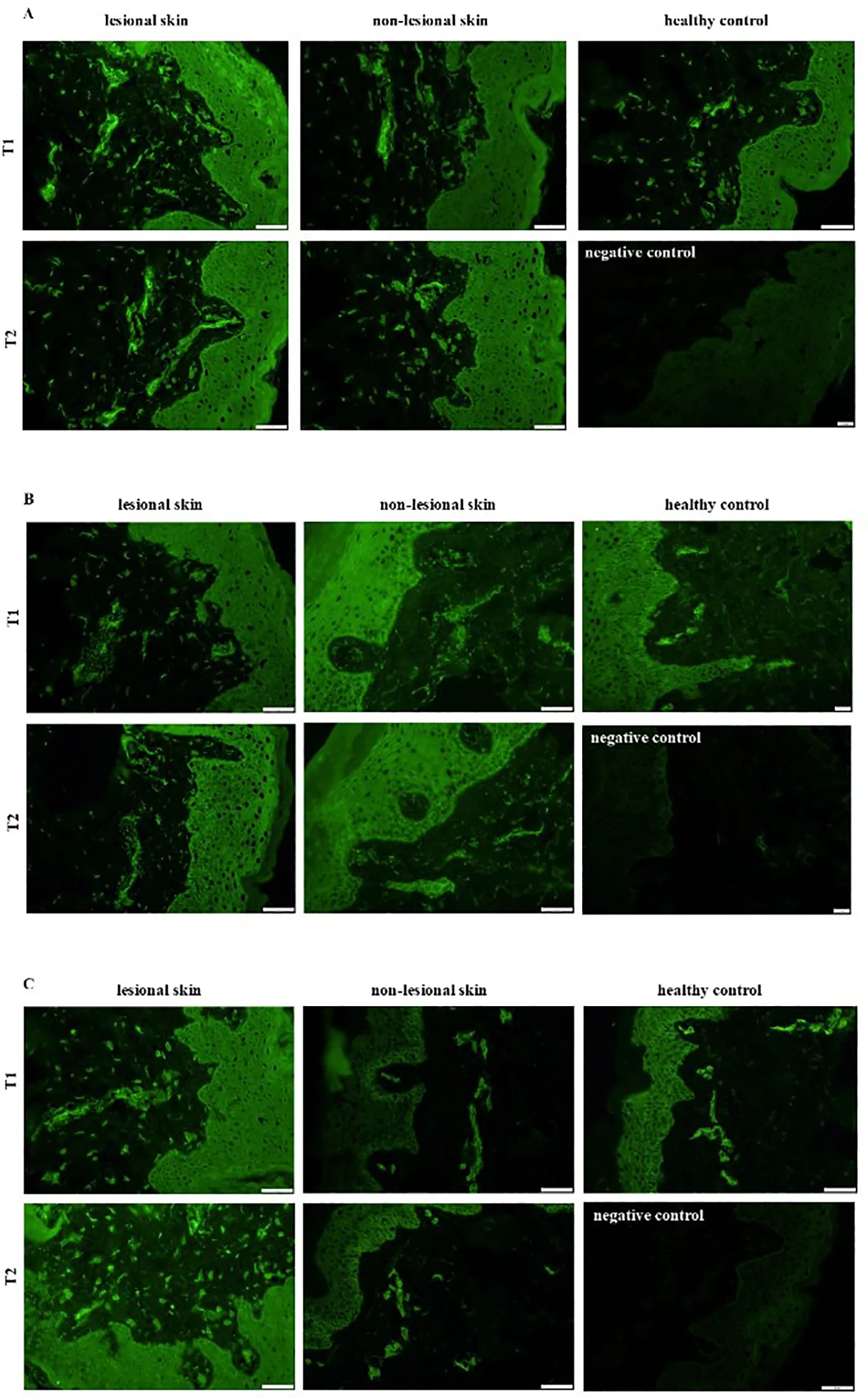
The representative sections of skin localization of **(A)** myeloperoxidase (MPO), **(B)** peptidylarginine deiminase 4 (PAD-4) and **(C)** DNase I in lesional (n=17) and non-lesional (n=7) skin of the patients with vitiligo before (T1) and after 6 months of laser therapy (T2) in comparison to the control group (n = 12). Negative control - without primary antibody. The proteins are marked in green (fluorescein). Magnification: 500x.

Analysis of protein expression in lesional skin at baseline revealed significantly elevated levels (p<0.05) of MPO (3,42 + 0.71), PAD-4 (3.45 + 0.56) and DNase I (3.42 + 0.56) compared with healthy volunteers (1.83 + 0.19 vs. 1.33 + 0.29 vs. 1.36 + 0.05, respectively) (**Figures 2A–C**, representative images are presented in **Figures 3A–C**). Following six months of therapy, only DNase I levels showed a significant change (T1 vs T2, 3.42 + 0.53 vs. 5.16 + 0.67; **Figure 2C**, representative images are presented in **Figure 3C**). The remaining factors showed no significant deviation from either baseline (T1) or control levels. In perilesional skin (the area surrounding the lesion), MPO and DNase I levels were comparable to those of healthy volunteers (**Figures 2A, C**, representative images are presented in **Figures 3A–C**); however, PAD-4 expression was already significantly elevated (p<0.05, **Figure 2B**, representative images are presented in **Figure 3B**) at baseline (2.38 + 0.49 vs. 1.33 + 0.29). The six-month therapy did not significantly alter the levels of the analyzed proteins in the perilesional skin (**Figures 2A–C**, representative images are presented in **Figures 3A–C**).

## 3 Material and methods

### 3.1 Study group

The study group included 17 adult patients (age >18 years) diagnosed with stable non-segmental vitiligo, treated at the Clinic and Department of Dermatology, Sexually Transmitted Diseases, and Clinical Immunology in Olsztyn, Poland. Exclusion criteria included malignancies, severe systemic diseases, acute infections, pregnancy, and lactation. The control group consisted of 31 healthy volunteers with no personal or family history of inflammatory or autoimmune diseases. The study protocol was approved by the Bioethical Committee of the University of Warmia and Mazury in Olsztyn (Resolution No. 7/2026). Informed consent was obtained from all participants. Disease activity was assessed at baseline by a dermatologist using the Vitiligo Area Scoring Index (VASI) and Body Surface Area (BSA). The clinical characteristics of the patients are presented in **Table 2**. All patients included in this retrospective analysis underwent a six-month washout period, during which no topical or systemic treatments were used, to ensure data reliability.

**Table 2:** Clinical patient parameters.

Vitiligo (n=17)	Age (years)	C-reactive protein (CRP, mg/l)	Neutrophils (10^3/ul)	Diseases activity	
				VASI	BSA (%)
mean ± SEM	48.35 ± 3.1	1.95 ± 0.25	4.33 ± 0.39	3.03 ± 0.64	3.53 ± 0.71
median	47	1,8	4.33	3	3.5
min/max	28/71	0.6/3.8	2.11/8.69	0.46/10.35	0.5/11.5

### 3.2 Laser and narrowband ultraviolet B phototherapy therapy

In all cases, FCO2 laser therapy was administered—either as a monotherapy or in combination with NB-UVB phototherapy—at monthly intervals. Standardized laser settings were maintained throughout the study: 25 W power, 25 mJ pulse energy, 27% density, and a single-pulse mode. For patients receiving combination therapy, NB-UVB (311 nm) phototherapy was administered three times weekly.

### 3.3 Blood

Venous blood samples (6 mL) were collected before treatment (T1) and after 6 months (T2) using Vacuette tubes (Greiner Bio-One GmbH, Austria). For the study group (n=17), samples underwent hematological and biochemical analyses, including neutrophil counts and C-reactive protein (CRP) levels. These measurements were performed at the Laboratory of the Municipal Polyclinical Hospital in Olsztyn using Mindray BC 760CS (Cormay Diagnostics) and Cobas Pure (Roche Diagnostics) analyzers. Serum levels of MPO-DNA complexes, DNase I, and PAD-4 were assessed by enzyme-linked immunosorbent assay (ELISA) using commercially available kits (Enlibio, China, and Biorbyt, UK). The detection ranges were 0.156–20 ng/mL for MPO-DNA, 39.07–5000 pg/mL for DNase I, and 0.32–20 ng/mL for PAD-4. Analytical procedures were conducted strictly according to the manufacturer’s protocols. The validity of the assays was confirmed by demonstrating parallelism between the standard curve and dilutions of randomly selected serum samples. The intra- and inter-assay coefficients of variation were <8% and <12%, respectively. The assay sensitivity—defined as the lowest concentration differentiable from zero—was determined to be 0.06 ng/mL for MPO-DNA, 30 pg/mL for DNase I, and 0.121 ng/mL for PAD-4. Absorbance was measured at 450 nm using a Multiskan FC microplate reader (Thermo Fisher Scientific, Waltham, MA, USA).

### 3.4 Tissue

The assessment of MPO, PAD-4, and DNase I protein expression in biopsy specimens was performed by immunofluorescence, following previously described methods with modifications (16). Four 4-mm punch biopsies were obtained per patient under local anesthesia (1% lignocaine): from lesional skin (n=17) and perilesional (non-lesional) skin (n=7) in two time points (T1 and T2 – after 6 months). Additionally, biopsies were collected from healthy volunteers (n=12) as healthy controls. Tissue samples were cut into 4-um-thick sections using a cryostat (SLEE MEV, Nieder-Olm, Germany) and mounted onto glass slides coated with poly-L-lysine (Menzel-Glaser, Braunschweig, Germany). Sections were fixed in acetone for 10 min and rinsed in 0.01 M PBS. To minimize non-specific binding, sections were incubated with 2.5% normal horse serum (Vector Laboratories, Burlingame, CA, USA) for 30 min at room temperature. Subsequently, samples were incubated overnight at 4 °C with rabbit anti-MPO, anti-PAD-4, and anti-DNase I polyclonal antibodies (1:50; Merck Millipore, Billerica, MA, USA). The following day, sections were washed in PBS and incubated for 30 min with secondary antibodies (ImmPRESS Universal Anti-Mouse/Rabbit Ig reagent; Vector Laboratories). For negative controls, primary and/or secondary antibodies were replaced with 0.01 M PBS. Immunofluorescence was evaluated using a fluorescent microscope (CH30/CH40; Olympus, Tokyo, Japan). Image analysis was performed according to the method described by Kasprowicz-Furmańczyk et al. (16). The immunoreactivity of each protein was quantified using ImageJ software; a threshold was applied to select the gray-value range corresponding to positive staining. For statistical analysis, percentage data were subjected to arcsine transformation.

### 3.5 Statistical analysis

After checking the assumptions of normality and homogeneity of variances, the results were analyzed using non-parametric tests (the data were not normally distributed). The Wilcoxon signed-rank test was used to compare two time points, and the Kruskal-Wallis test was used to compare each of the studied groups with the control group. Spearman’s test was performed to assess the correlation between factors. All statistical analyses were performed using Statistica software (ver. 13, StatSoft Inc., Tulsa, OK, USA). Differences were considered statistically significant at p < 0.05. The results are presented as means ± SEM in the table and as medians with interquartile ranges (IQR) in the figure.

## 4 Discussion

The present study provides evidence that vitiligo is associated with compartment-specific differences in neutrophil-associated markers and extracellular DNA-related pathways. Distinct patterns of the analyzed markers were observed in the circulation, lesional skin, and clinically unaffected perilesional skin, suggesting differences in the molecular and inflammatory characteristics of these compartments. Importantly, the observed changes should be interpreted as alterations in NET-related pathways rather than direct evidence of neutrophil extracellular trap formation.

In the systemic compartment, patients with vitiligo exhibited increased circulating MPO–DNA complexes together with elevated serum DNase I levels. Although MPO–DNA complexes are widely used as a surrogate marker of NET-associated material, their presence does not necessarily indicate active NET formation. Rather, increased circulating MPO–DNA complexes may reflect enhanced neutrophil activation, the release of extracellular DNA–protein complexes from inflamed tissues, or disturbances in extracellular DNA homeostasis. Similarly, the elevated serum concentration of DNase I may represent a compensatory response to increased extracellular DNA availability. However, because only circulating DNase I concentrations were assessed, these findings should not be interpreted as evidence of increased enzymatic activity or enhanced NET clearance. Functional studies evaluating DNase I activity will be required to clarify its role in extracellular DNA homeostasis in vitiligo.

In contrast to MPO–DNA complexes and DNase I, circulating PAD-4 concentrations were reduced compared with healthy controls and remained unchanged following treatment. This finding suggests that serum PAD-4 may not adequately reflect local tissue events and raises the possibility that PAD-4-associated processes are regulated differently in the circulation and within the cutaneous microenvironment. Taken together, these observations suggest that the systemic profile observed in our patients is more consistent with altered extracellular DNA homeostasis than with generalized activation of NETosis.

The cutaneous findings revealed a distinct pattern. Before treatment, lesional skin showed increased expression of MPO, PAD-4, and DNase I compared with healthy skin. The parallel increase in proteins involved in extracellular DNA release and degradation suggests enhanced extracellular DNA turnover within active vitiligo lesions. However, these observations should not be interpreted as direct evidence of NET formation, as the presence of NET structures was not demonstrated. Instead, they indicate local dysregulation of neutrophil-associated pathways within the inflammatory milieu of vitiligo lesions. Whether these alterations participate in melanocyte damage, reflect secondary responses to ongoing inflammation, or represent compensatory mechanisms regulating extracellular DNA clearance remains unresolved.

One of the most intriguing observations of the present study was the molecular profile of clinically unaffected perilesional skin. While MPO and DNase I expression remained comparable with healthy controls, PAD-4 expression was significantly increased despite the absence of visible depigmentation or other changes in the evaluated markers. This finding suggests that molecular alterations may precede clinically detectable skin involvement. These observations support the concept that clinically normal-appearing skin may already harbor early immunological changes before overt depigmentation becomes evident. Although PAD-4 is widely recognized for its role in chromatin citrullination during NET formation, accumulating evidence indicates that it also participates in broader inflammatory and nuclear regulatory processes. Therefore, isolated PAD-4 upregulation in perilesional skin may reflect early local immune dysregulation rather than established NET activation. Notably, these changes persisted after six months of therapy, suggesting that subtle molecular abnormalities within clinically unaffected skin may remain despite improvement of active lesions.

**Figure 4:**
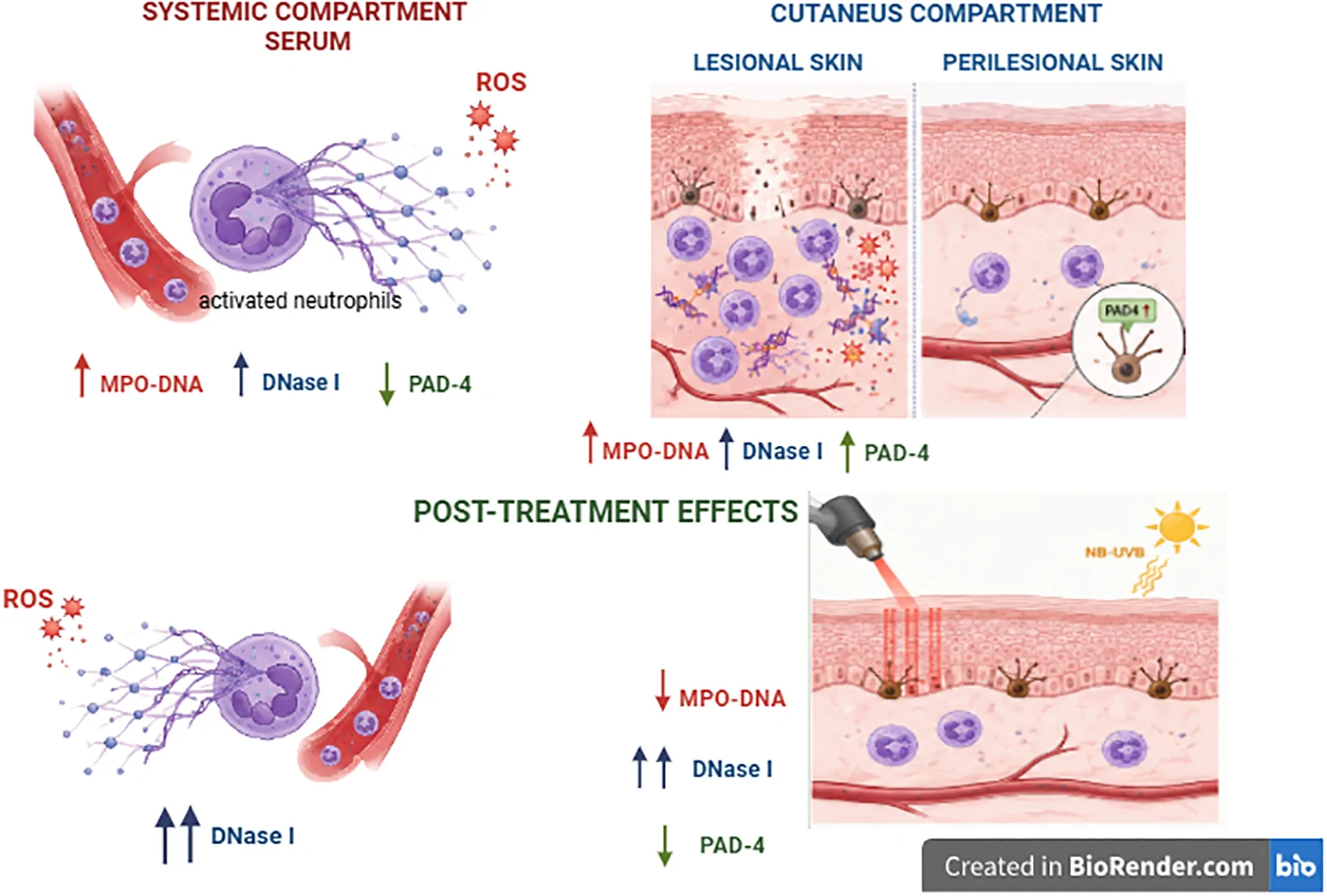
Schematic representation of compartmentalized alterations in NET-related markers in vitiligo before and after fractional CO_2_ laser therapy.

Following combined fractional CO_2_ laser and NB-UVB therapy (**Figure 4**), MPO and PAD-4 expression decreased in lesional skin, whereas DNase I increased in both lesional skin and serum. These findings suggest that treatment is associated with modulation of neutrophil-associated pathways and extracellular DNA turnover. However, because fractional CO_2_ laser induces controlled epidermal injury followed by tissue remodeling, the observed molecular changes may reflect not only disease-related immune regulation but also biological processes involved in wound healing and tissue repair. As both therapeutic modalities were administered concurrently, the relative contribution of fractional CO_2_ laser and NB-UVB to the observed immunological changes cannot be determined.

Growing evidence indicates that complex interactions between adaptive and innate immune responses, oxidative stress, and dysregulated tissue homeostasis characterize vitiligo. While autoreactive CD8^+^ T cells remain central mediators of melanocyte destruction, increasing attention has been directed toward the contribution of innate immune mechanisms to disease initiation and progression. Infiltration of cytotoxic CD8^+^ T cells into vitiligo lesions, together with alterations in regulatory T-cell populations, has been consistently associated with melanocyte-directed immunity and altered expression of immune-associated molecules and transcriptional regulators, including CCL17, CCR4, Tbx21, Eomes, and Blimp1 (4, 17, 18). In parallel, single-cell transcriptomic analyses of peripheral immune cells have revealed altered immune cell states, including activation signatures in neutrophils, monocytes, and B cells, supporting the concept that both innate and adaptive immune responses participate in the inflammatory network underlying vitiligo (19).

Oxidative stress represents another key component of vitiligo pathogenesis and provides a potential mechanistic link between melanocyte injury and extracellular DNA metabolism. Reactive oxygen species generated by activated neutrophils, macrophages, and damaged tissue contribute to oxidative injury to melanocytes, which are particularly susceptible to oxidative stress due to their high metabolic activity and relatively limited antioxidant capacity (20, 21). Cellular injury induced by oxidative stress may increase the release of extracellular nucleic acids, thereby activating DNA clearance pathways independently of classical NET formation. Within this context, the elevated circulating DNase I concentrations observed in our study may reflect enhanced extracellular DNA turnover rather than NET-specific degradation. However, because only circulating DNase I concentrations were measured, these findings should not be interpreted as evidence of increased enzymatic activity, and functional studies will be necessary to determine the biological significance of this observation (22, 23).

Further support for the involvement of innate immune pathways comes from studies investigating calprotectin, a damage-associated inflammatory protein predominantly expressed and released by activated neutrophils and monocytes. Increased calprotectin levels have been reported in patients with vitiligo and have been linked to cytokine production, oxidative stress, and amplification of local inflammatory responses (9, 10). Together with our findings, these observations support the concept that neutrophil-associated mechanisms may contribute to the inflammatory milieu of vitiligo, although their precise role remains to be established.

Only a limited number of studies have examined NET-related markers in vitiligo, and the available evidence remains inconsistent. In our cohort, circulating MPO–DNA complexes and DNase I concentrations were increased, whereas serum PAD-4 concentrations were reduced. Rather than supporting uniform activation of NET-related pathways, this profile suggests differential regulation of extracellular DNA release and clearance across systemic and tissue compartments. Previous studies in vitiligo have mainly evaluated MPO-related parameters, reporting reduced plasma MPO concentrations, no association between MPO gene polymorphisms and disease susceptibility, or increased MPO levels following phototherapy (24–26). Direct comparisons with the present study are therefore difficult because of differences in study design, patient populations, and the biomarkers evaluated.

Genetic evidence also suggests that pathways involved in extracellular DNA regulation may be relevant to vitiligo susceptibility. A large genetic study involving more than 1,000 patients identified an association at the rs1408944 locus within a DNase-hypersensitive region, while functional analyses implicated ZMIZ1 and other genes previously linked to vitiligo pathogenesis (27). Although these findings do not establish a direct relationship between DNase I function and disease development, they support the concept that extracellular DNA regulation may represent one component of the complex molecular network involved in vitiligo.

Interestingly, despite the differences observed in neutrophil-associated markers between patients and healthy controls, we found no significant associations between the analyzed markers and clinical measures of disease severity, including VASI and BSA. Likewise, conventional systemic inflammatory parameters, including neutrophil count and CRP concentration, remained comparable with those observed in healthy controls. These findings indicate that the analyzed markers cannot currently be considered indicators of disease activity. Instead, they may reflect biological processes involved in vitiligo pathophysiology and treatment-associated tissue responses, although their biological and potential clinical significance requires further investigation in larger longitudinal studies.

Taken together, these observations suggest that systemic alterations are more consistent with changes in extracellular DNA homeostasis than with generalized activation of NETosis. However, the present study demonstrates associations rather than causality. Whether the observed molecular changes contribute directly to melanocyte damage, arise secondary to ongoing inflammation, or represent compensatory mechanisms involved in extracellular DNA homeostasis remains to be determined.

Several limitations should be considered when interpreting our findings. First, the relatively small sample size may have limited the statistical power to detect more subtle associations, particularly between NET-related markers and clinical measures of disease severity. Consistent with this, no significant correlations were identified between the analyzed markers and VASI or BSA scores, indicating that these markers cannot currently be regarded as reliable indicators of clinical activity. Second, vitiligo is a multifactorial disorder involving complex interactions between adaptive immunity, innate immune responses, oxidative stress, and genetic susceptibility. Consequently, the evaluated proteins in this study represent only one aspect of the disease’s immunopathogenesis.

Third, detailed information regarding previous therapeutic interventions was not available for all participants. Therefore, the potential influence of prior treatments on circulating and cutaneous neutrophil-associated markers cannot be excluded.

An additional limitation arises from the study design. All patients received combined fractional CO_2_ laser and NB-UVB therapy, precluding assessment of the independent effects of each treatment modality. Because fractional CO_2_ laser induces controlled tissue injury followed by wound healing and remodeling, some of the observed changes in NET-related markers may reflect treatment-associated regenerative processes rather than disease-specific immunological mechanisms (28). Furthermore, the absence of an untreated vitiligo comparator group limits the ability to distinguish therapy-induced changes from the natural temporal variability of immune responses associated with vitiligo. Future studies incorporating untreated patients, as well as laser-only and NB-UVB-only treatment groups, will be essential to define the specific contribution of each therapeutic intervention to the modulation of neutrophil-associated pathways.

Another important consideration is that this study evaluated circulating concentrations of DNase I rather than its enzymatic activity. Consequently, the observed increase in serum DNase I should not be interpreted as evidence of enhanced extracellular DNA degradation or NET clearance. Functional assays assessing DNase I activity will be necessary to clarify the biological significance of this finding.

Finally, the six-month follow-up period does not permit assessment of the long-term dynamics or prognostic significance of NET-related molecules. In addition, patients were not stratified according to different clinical phenotypes of vitiligo. Larger multicenter longitudinal studies incorporating diverse disease subtypes and mechanistic analyses will be required to validate these observations and determine their potential clinical relevance.

## 5 Conclusion

This study provides evidence that vitiligo is associated with compartment-specific alterations in neutrophil-associated markers and extracellular DNA-related pathways. Distinct molecular profiles identified in the circulation, lesional skin, and clinically unaffected perilesional skin suggest that systemic and local immune responses are differentially regulated during the course of the disease.

The observed modulation of these molecules following combined fractional CO_2_ laser and NB-UVB therapy suggests that neutrophil-associated pathways may be influenced during treatment; however, the individual contribution of each therapeutic modality remains unclear. Importantly, the present study demonstrates associations rather than causality, and the biological significance of the observed NET-related marker profile remains to be established. Further longitudinal and mechanistic studies are required to determine whether these alterations contribute to vitiligo pathogenesis, have prognostic value, or may represent potential biomarkers or therapeutic targets.

## Data Availability

The raw data supporting the conclusions of this article will be made available by the authors, without undue reservation.
