# Supplementary material for: Neutrophil-associated extracellular DNA pathways in vitiligo: compartment-specific changes following combined fractional CO2 laser and NB-UVB therapy
Source: Front Immunol. 2026 Sep 1;17:1897885. doi: 10.3389/fimmu.2026.1897885 (PMC13575744; doi:10.3389/fimmu.2026.1897885)

Supplementary Figure S1. Individual patient changes in clinical disease activity following 6 months of treatment. Each line represents an individual patient.

(A) Individual Vitiligo Area Scoring Index (VASI) values at baseline (T1) and after 6 months of treatment (T2).

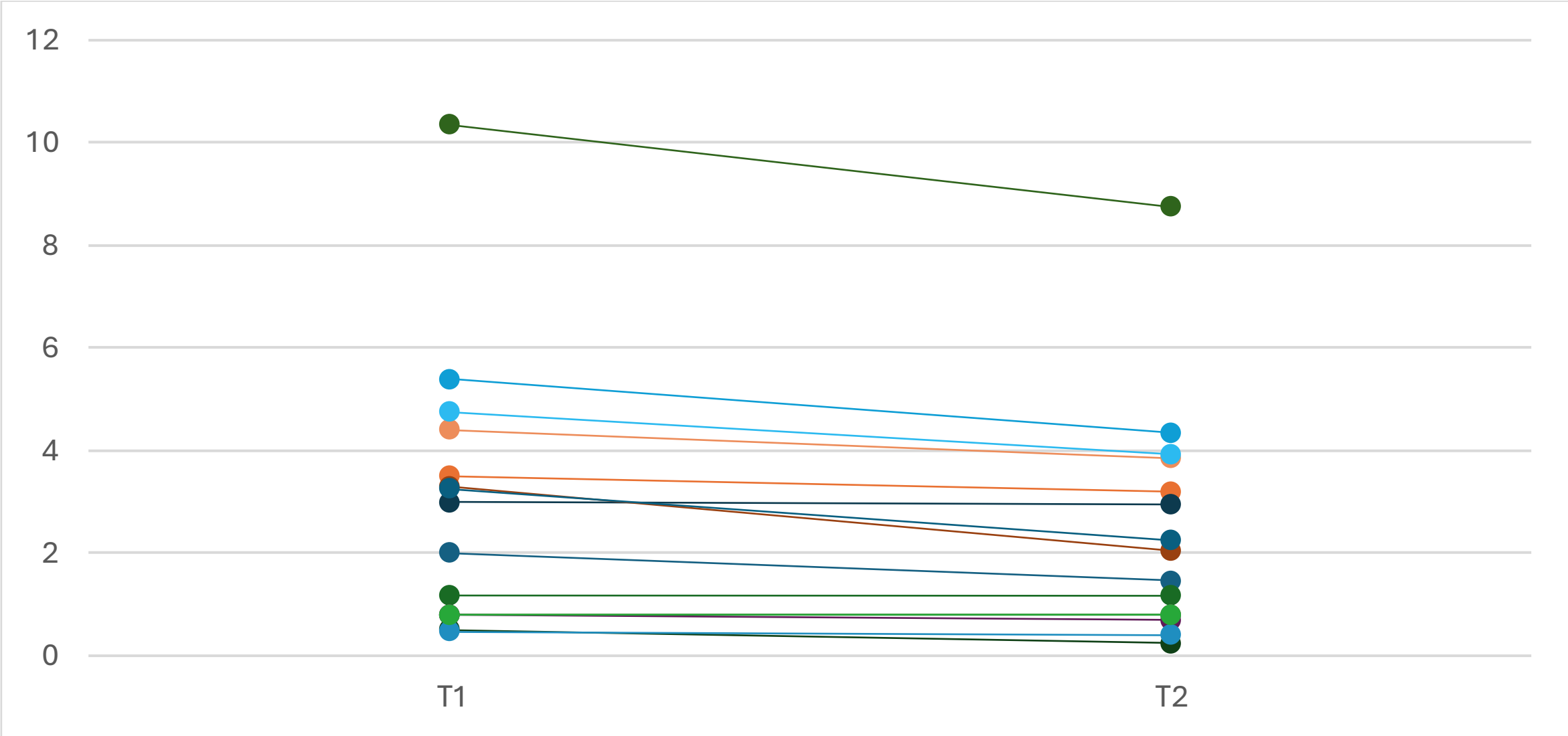

(B) Individual Body Surface Area (BSA) values at baseline (T1) and after 6 months of treatment (T2).

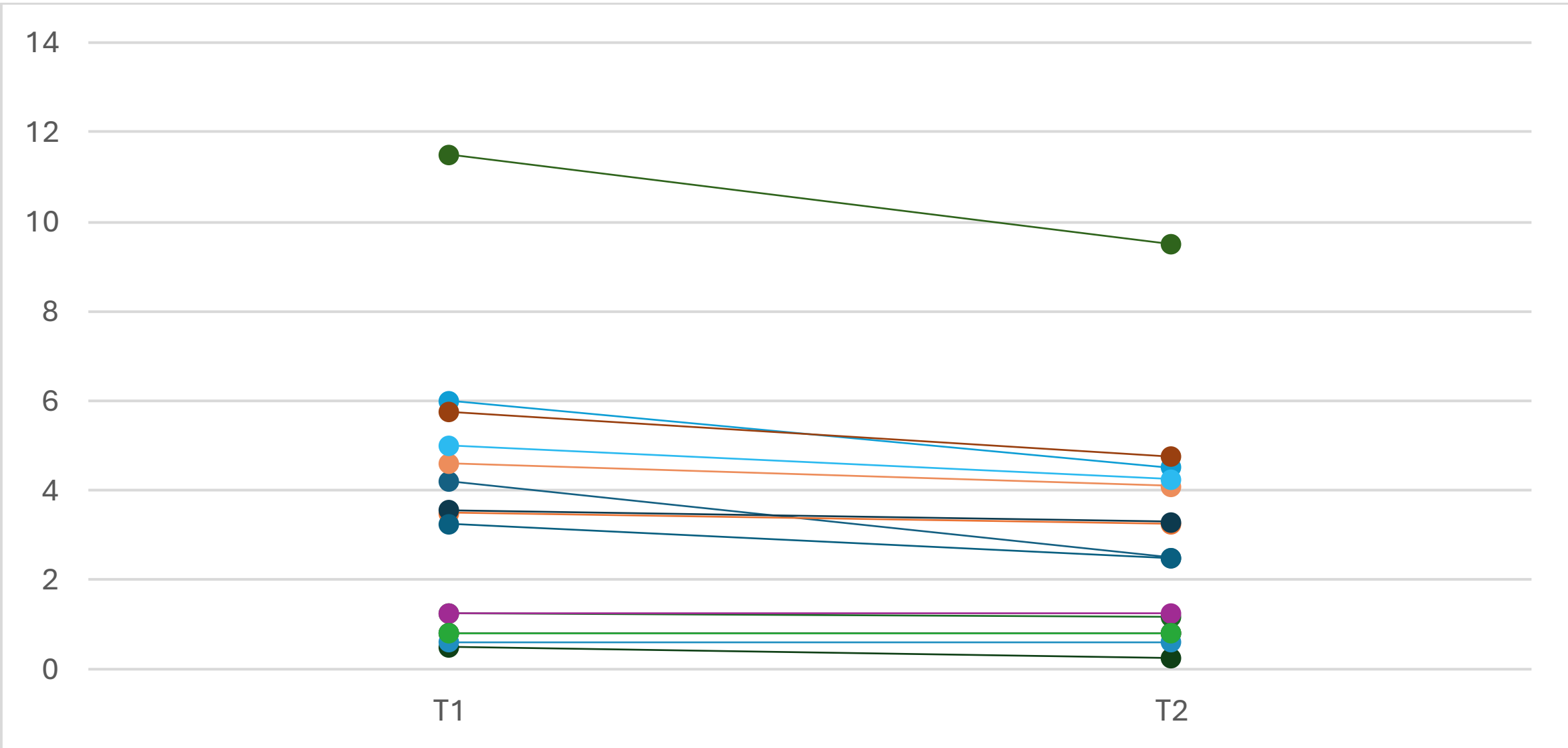

Supplement: Supplementary file 1 [file DataSheet1.pdf]
