# Supplementary material for: Neutrophil-associated extracellular DNA pathways in vitiligo: compartment-specific changes following combined fractional CO2 laser and NB-UVB therapy
Source: Front Immunol. 2026 Sep 1;17:1897885. doi: 10.3389/fimmu.2026.1897885 (PMC13575744; doi:10.3389/fimmu.2026.1897885)
Supplement: Supplementary file 2 [file DataSheet2.pdf]

Supplementary Figure S2. Individual baseline values (T1) of systemic inflammatory markers in patients with vitiligo and healthy controls.

	Study group		Control group	
	Neutrophil counts (× $10^3/\mu L$)	CRP (mg/L)	Neutrophil counts (× $10^3/\mu L$)	CRP (mg/L)
1.	3,11	2,8	4,56	0,6
2.	5,17	2,2	5,82	1,39
3.	8,69	3,4	6,3	0,86
4.	4,33	0,6	4,01	0,6
5.	4,57	1,2	4,16	0,97
6.	3,43	1,8	4,1	0,6
7.	4,33	1	1,98	0,6
8.	2,98	0,6	6,05	0,6
9.	4,89	3,8	3,14	0,67
10.	2,62	1,1	2,3	1,63
11.	2,98	1,7	4,01	0,64
12.	3,45	2,8	2,36	0,97
13.	3,98	2,1	2,98	0,6
14.	2,11	0,8	3,97	0,6
15.	4,56	1,2	4,14	1,27
16.	6,23	3,4	4,05	0,6
17.	6,14	2,6	2,66	1,23
18.			1,98	0,6
19.			5,45	0,6
20.			2,56	0,6
21.			3,09	0,6
22.			3,24	1
23.			4,04	0,66
24.			4,16	0,6
25.			4,56	0,6
26.			5,82	1,39
27.			6,3	0,86
28.			4,01	0,6
29.			4,16	0,97
30.			4,1	0,6
31.			1,98	0,6
